# Education aimed at increasing international collaboration and decreasing inequalities

**DOI:** 10.1002/1878-0261.12460

**Published:** 2019-02-20

**Authors:** Ingemar Ernberg

**Affiliations:** ^1^ Department of Microbiology, Tumor Biology and Cell Biology Karolinska Institutet Stockholm Sweden

**Keywords:** cancer prevention, Comprehensive Cancer Center, dissemination, higher education, translational cancer research

## Abstract

Educational initiatives in cancer research have to align with the needs of patients, individuals at risk, healthcare systems and public health organisations. The above interests demand strong translational interactions between basic research, clinical/prevention research and entrepreneurship. The resulting synergy between these three entities is expected to stimulate identification of unresolved issues in cancer biology, as well as unmet needs in diagnostics, treatment and prevention. It will also encourage the development of international research collaborations and, in turn, improve access to innovative research infrastructures. Education and dissemination of knowledge and technologies must be a cornerstone of any future European mission‐oriented approach to cancer, as it will ensure that new cancer treatments reach all patients within the European Union, and also help reduce gross inequalities in cancer incidence and mortality. A large number of educational institutions ranging from local universities to pan‐European organisations have developed excellent educational activities. However, a cancer mission will highlight additional roles for higher education that will complement and provide novel approaches. Educational and training activities should target the general public (dissemination) for primary cancer prevention, as well as the next generation of cancer researchers in basic and clinical research all over Europe. The experiences of patients are also needed to improve health‐related quality‐of‐life and outcomes research. A mission approach to cancer would enhance the exchange of researchers within Europe and worldwide, and prioritise collaborations between Western/Central and Eastern Europe countries. The Comprehensive Cancer Centres (CCCs) will be crucial to train scientific staff in established centres as well as in candidate centres aspiring to join networks of CCCs. In addition, CCCs will have an important role to play by offering educational programmes for the next generation of clinical/research leaders.

**Abbreviations**
CCC: Comprehensive Cancer Centre
CPE: Cancer Prevention Europe
EACR: European Association for Cancer Research
ECPC: European Cancer Patient Coalition
EORTC: European Organisation for Research and Treatment of Cancer
EU: European Union
OECI: Organization of the European Cancer Institutes

## 1 New demands on higher education in a mission approach to cancer

As described in other chapters in this issue, cancer is an increasing problem not only in the European Union (EU) countries, but also globally (see also Ferlay *et al*., 2018; Forman *et al*., 2018). Research is needed to target these negative trends, focusing on innovation within therapeutics and prevention. Achieving this will require a coherent cancer research continuum in order to translate new scientific information to patients and at‐risk individuals.

Cancer is one of the most complex medical disciplines, both from a clinical and a research point of view. As a result, education is fragmented, with a number of action areas and actors. Research is pursued in a large number of diverse areas, each linked to educational needs. Multidisciplinary cancer treatment and care must be supported by educational activities in order to deliver high‐quality treatment and care.

However, this is an issue primarily for the healthcare systems. For a mission‐oriented approach to cancer, translational research is vital since the innovations in therapeutics and prevention will be the prime targets (Celis and Pavalkis, 2017; see also article by Celis and Heitor 2019). Therefore, education as a component of a mission has to (a) focus on the increased quality of research and on a coherent cancer research continuum, (b) accommodate the expansion of research collaborations in the EU countries and (c) decrease existing inequalities both within and between countries. This will not be possible using traditional educational courses/events alone, but will require new ideas to make education available for young researchers within the EU, as well as by expanding research collaborations to include educational outcomes as a goal.

## 2 Current educational activities in cancer research in Europe

**Figure 1:**
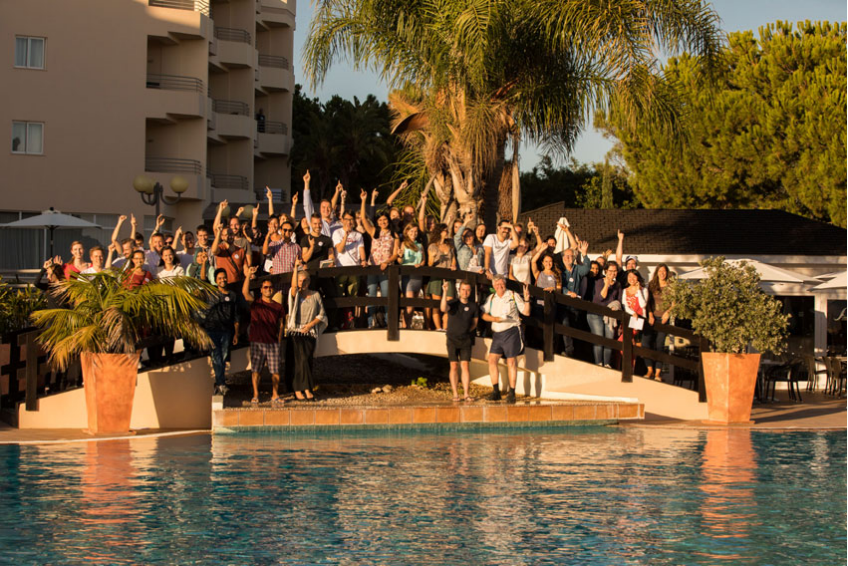
Cancer Core Europe Summer School, Algarve, Portugal, October 2018. Photograph of the 70 participants (doctoral students, post docs and clinicians) and some of the teaching faculty, including the author in the first row to the right.

A number of European organisations play important roles in both clinical and research education through congresses, practical courses, workshops and publications; examples include European Society for Medical Oncology (ESMO), European Society for Radiotherapy and Oncology (ESTRO), European Society of Surgical Oncology (ESSO), International Society of Paediatric Oncology (SIOP), European School of Oncology (ESO) and European Association for Cancer Research (EACR). The European Molecular Biology Laboratory (EMBL) provides training for young researchers, whilst The European Molecular Biology Organization (EMBO) organises courses and supports young researchers with fellowships. The Federation of European Biochemical Societies (FEBS) also provides fellowships, supports courses and arranges annual FEBS congresses. The European Oncology Nursing Society (EONS) focuses on education and training of nurses, with the aim of innovating the field of supportive care. The main mission of the European Organisation for Research and Treatment of Cancer (EORTC) is to support clinical research collaboration. By working in an EORTC group, members are kept updated in clinical trial methodology. Moreover, EORTC supports education in research, with advanced and comprehensive courses for high‐quality clinical trials. The Organisation of European Cancer Institutes (OECI), having a focus on development of Comprehensive Cancer Centres (CCCs), arranges annual meetings with educational content, mainly focusing on structures and activities of CCCs, such as molecular pathology, outcomes in research structures and health economics. The EurocanPlatform survey of research education activities in 23 centres (including the Cancer Core Europe centres) in 2011 showed a large number of educational events representing the majority of research areas, but there were no educational events with a focus on translational cancer research. Therefore, the Annual Summer School on Translational Cancer Research was established within the EurocanPlatform project, which is now a sustainable educational event under the Cancer Core Europe umbrella. In collaboration with Cancer Prevention Europe (CPE), translational research for prevention is represented. The Summer School is open to young researchers in all countries and has supported researchers from centres with restricted economic resources by providing fellowships (Fig. 1).

Most European cancer centres are part of university hospitals, where the responsibility for clinical activities is separated from responsibility for activities in basic/preclinical research departments. One way to bridge the gap between preclinical and clinical research is by the establishment of MD/PhD programmes. This facilitates multidisciplinary research collaboration and supports the development of translational cancer research.

An important goal of a mission‐based approach to cancer is to increase the level of knowledge in all EU countries in order to stimulate collaborations and decrease inequalities. Therefore, large centres should be more open towards offering opportunities for young researchers from all member states to participate in in‐house research educational programmes.

## 3 Exchange of researchers and bilateral collaborations between Western/Central and Eastern Europe

Exchange of researchers is an important way to develop and stimulate education, as it gives young researchers the opportunity to learn new technologies, visit new research environments and initiate collaborations. Enhancing research collaborations between centres and countries may lead to decreased inequalities. Exchange of researchers is facilitated by the development of consortia of cancer research centres (e.g., Cancer Core Europe and CPE), and in discussion of a mission‐oriented cancer research has resulted in an agreement to increase the number of consortia to cover various research geometries and different components of the research continuum (Berns, 2019). Further, if a mission on cancer becomes a reality, there will be a portfolio of projects available for all cancer researchers. Evaluation of applications, based on excellence, will stimulate the development of new international research collaborations and also increase the opportunities for exchange of researchers.

Bilateral institutional collaborations are an innovative way to support research and education, and also decrease inequalities. Well‐developed centres in Western/Central Europe could establish formal collaborations with centres in Eastern Europe. Here, the recently formed collaboration between the German Cancer Research Centre/National Centre for Tumour Diseases in Heidelberg and the new cancer centre in Athens is a good example. This type of collaboration, aimed at supporting centres in Eastern Europe, has already been suggested by the leadership of Cancer Core Europe and represents a way to stimulate and support research and education and decrease inequalities. Provided it is well planned, it should be of added value for not only the collaborating centres, but also for research collaboration between the EU countries as a whole.

## 4 Role of Comprehensive Cancer Centres in education

Organisation of multidisciplinary cancer treatment/care, prevention, research and education and the concept of the CCC are ways to assure high‐quality research environments and to bridge the gap with the healthcare area (Oberst, 2019). OECI and German Cancer Aid provide educational activities on organisational aspects and offer methodologies to designate CCCs. Establishment of CCCs has a strong educational impact, on both clinical activities and research. Currently, consortia of CCCs are being developed to reach the critical mass necessary for innovative translational research. Based on international criteria, the accreditation methodology will be helpful to reduce inequalities not only regarding research, but also cancer care, prevention and education. Furthermore, work is ongoing to define a role of the CCCs within a geographical outreach area in quality control of cancer care and prevention, and to involve surrounding clinics in research and education (Brandts, 2019). With a formalised part of the accreditation methodology focused on outreach, new dimensions of education will be introduced that reach clinics and patients outside CCCs, as well as the general public, in order to facilitate innovation and decrease inequalities.

The increasing demands on infrastructure and organisation of translational cancer research require that a designation programme for CCCs of Excellence (DoE) be established under the umbrella of the EACR (Berns, 2019; Ringborg *et al*., 2018). The programme is helpful in increasing the quality of CCCs that have a primary focus on translational research. With the development of consortia of CCCs to increase critical mass and Open Science, the DoE programme with defined criteria for translational cancer research is also important for quality control of the consortia. This programme has a strong educational impact and aims to continuously improve the strategies and infrastructure for translational cancer research.

## 5 Educational programmes for the next generation of clinical/research leaders

We cannot ignore the need to recruit and train the next generation of clinical science leaders of translational cancer research in the European CCCs. The goal will be to further develop the next generation of translational cancer research and treatment. The Oncology curriculum is of variable quality all over Europe, and there is a consistent difficulty for young clinical researchers to keep up with new techniques, knowledge and opportunities. In some countries, young oncologists lack insight into modern cancer biology and opportunities offered by new technologies, for example, high‐throughput technologies and systems biology, hampering their ability to take the lead. This can only be remedied by a joint effort by several major top centres to train young clinical researchers. These would ensure the critical mass of teachers, scientists and technical competence required to lift all of Europe into the future of cancer treatment.

To this end, Cancer Core Europe plans to start such an advanced training programme in 2019, which will run for 3 years with annual cohorts of around 30 carefully selected young clinical cancer researchers. The participants will receive a tool‐kit, knowledge and techniques for pursuing cutting‐edge translational research; develop a strong network among the participants at the European level; and acquire clear insight into the different capabilities, strengths and weaknesses of each centre.

## 6 Health‐related quality‐of‐life and outcomes research include the patients’ perspective

A mission approach to cancer research aims at tackling cancer as a societal challenge. Research needs to innovate across all aspects of the clinical pathway. An important missing piece is outcomes research for prevention and therapeutics, a prerequisite for constructive health economics research. Furthermore, there is a need to expand research on health‐related quality of life, which is one of the important outcomes of treatment together with survival benefit. Of special interest is research linked to rehabilitation, psychosocial oncology, and supportive and palliative care. This includes cancer survivorship, which is an increasing human and medical challenge due to the growing number of surviving patients with long‐term side effects of a cancer diagnosis and treatment. As health‐related quality‐of‐life research is a multidisciplinary research area involving physicians, nurses, psychologists, physiotherapists and other professionals, it requires specific educational activities.

The European Cancer Patient Coalition (ECPC) plays an important role in the main parts of cancer research, care and prevention, by actively participating in a number of European projects to improve patient outcomes (De Lorenzo and Apostolidis, 2019). The more researchers, stakeholders in research and cancer care/prevention and patient organisations agree on problems, strategies and policy, the better the chances of influencing politicians. Education of patients and patient organisations should therefore be prioritised. Needless to say, the ECPC has a focus on inequalities of all types, both within and between the EU countries.

Education of the general public is also an important part of primary cancer prevention. For the most part, the general public are interested in cancer, and this interest should be supported by educational activities. For several areas of cancer research, increased knowledge will be followed by increased engagement by the general public, which might help to ensure that cancer research receives more public funding.

## 7 Conclusions

A future European mission‐oriented approach to cancer has to include broad educational activities. Numerous organisations and centres offer educational programmes in specific areas of basic/preclinical and clinical research. There is a need to strengthen education in translational cancer research for both therapeutics and prevention. Other unmet needs include education in prevention, outcomes research, health‐related quality of life, and health economics. Structuring cancer research in the EU towards a mission‐oriented approach to cancer will have important educational benefits. Ensuring that CCCs take responsibility for treatment/care, prevention, research and education will increase the educational level of all categories of staff. Educational activities will help ensure that the outreach programmes of CCCs will reach the general public, patients and clinics outside CCCs. The development of consortia such as Cancer Core Europe and CPE will promote research interactions between centres with important educational impact. From an EU perspective, research collaborations between centres in Central/Western and Eastern Europe will increase the exchange of researchers. Consortia of CCCs will play an important role in offering educational programmes for the next generation of clinical research leaders. Most importantly, structuring of educational activities on research and development will be important to decrease current inequalities.

## Conflict of interest

The author declares no conflict of interest.
